# Vegetation development and nutrients supply of trees in habitats with high sulfur concentration in reclaimed former sulfur mines Jeziórko (Southern Poland)

**DOI:** 10.1007/s11356-017-9638-5

**Published:** 2017-07-15

**Authors:** Justyna Likus-Cieślik, Marcin Pietrzykowski

**Affiliations:** Faculty of Forestry, Institute of Forest Ecology and Silviculture, Department of Forest Ecology and Reclamation, University of Agriculture in Krakow, al. 29 Listopada 46, 31–425, Krakow, Poland

**Keywords:** Plant nutrition, Reclamation, Reforestation, Sulfur contamination, Sulfur extraction by underground melting method, Frasch method

## Abstract

The paper presents an assessment of vegetation (composition and cover-abundance), nutrient supply, and especially sulfur accumulation in the trees foliage (birch and pine) used in reforestation and wood small-reed (*Calamagrostis epigejos* (*L.*) Roth) appearing in succession on reclaimed areas of the former Jeziórko sulfur mine (southern Poland, Tarnobrzeg region). In researched area, three categories of vegetation cover were determined: category D—degraded and unsuccessfully reforested plots, and two categories with successful reforestation: P—pine and B—birch stands. On each category, four study plots (4–6 areas each, depends on site category) were established. Soil and vegetation samplings (current year and 2-year-old pine needles, birch leaves, and wood small-reed foliage) were collected on the subplots established in regular grid square (10 × 10 m) in each category. Basic soil properties and nutrient content in soils and vegetation were analyzed. Trees grew well in areas where neutralization and reclamation treatments were carried out properly and showed a good supply of nutrients (exception of phosphorus and nitrogen), while on category D, only herbaceous vegetation with low cover-abundance and dominated by wood small-reed were noted. Linear correlations between the soil and trees nutrients content occurred, while the correlations between the soil and wood small-reed did not occur. Wood small-reed did not display increased sulfur uptake which may indicate a strategy of blocking pollutant uptake from the soil and may be recommended as a species resistant to sulfurous soils.

## Introduction

Sulfur is a macroelement which is necessary for the growth and proper development of plants, and its concentrations in plant foliage tissues indicating optimal growth usually range from 1000 to 5000 mg kg^−1^ (Tisdale et al. 1993; Marcshner 2012). On the other hand, sulfur and its compounds in excessive concentrations result in a degradation of the soil environment and exhibit phytotoxic properties (Knabe 1976; Kabata-Pendias et al. 1995; Feliciano et al. 2001; Rinne et al. 2010). The immediate impact of SO_2_ on vascular plants is demonstrated by negative effects on the root system, foliage, and in tissue damage (Rinne et al. 2010; Marcshner 2012; Wei et al. 2017). As a result of dust from industrial emissions which settles on leaves, plant stomata are clogged, and hence, the processes of photosynthesis and transpiration are disturbed (Feliciano et al. 2001; Wei et al. 2017). In the case of trees, this leads to a reduction of leaf number, thinning of tree crowns, deformed canopy shape, and limited growth (Tomlinson 1983). High sulfur content results in an increase of nutrient leaching into the soil profile, the displacement of base cations Ca^2+^ and Mg^2+^ from the sorption complex which eventually leads to soil acidity. This process increases the mobility of trace elements that are harmful firstly to soil organisms and which subsequently disturb biogeochemical cycles and vascular plant nutrition (Menz and Seip 2004). The natural sulfur content in the soils around the world varies and ranges from 100 to 1000 mg kg^−1^ (Kabata-Pendias et al. 1995; Stevenson and Cole 1999). In agricultural soils, sulfur content should not exceed 500 mg kg^−1^ (EMA 1996). In areas with elevated sulfur content, for example, near sulfur extraction and processing plants and in highly industrialized areas, sulfur content which is significantly higher than the figures cited here is frequently reported.

Anthropogenic contamination of soil with mineral sulfur on an unprecedented global scale occurs in former Frasch process sulfur mining areas. In the areas where the mineral was extracted using this method, there are vast areas which are difficult to reclaim and which are heavily contaminated by sulfur (Likus-Cieślik et al. 2017). In these areas, sulfur contamination occurs especially around the boreholes and along technological installations where liquid sulfur leaked out (Gołda 2003; Likus-Cieślik et al. 2017). One of the largest discovered sulfur deposits in the world (Piaseczno-Machów-Jeziórko-Jamnica deposit) is located in southern Poland in the vicinity of the town of Tarnobrzeg (Ober 2002; Michno et al. 2009). Until 2001, sulfur was extracted from Jeziórko mine using Frasch process. This method is still used in Poland in an operating Osiek mine in the same area of sulfur-bearing deposits (Gołda et al. 2005; Likus-Cieślik et al. 2017). Around the world, the borehole method has been used among others in the USA (until 2000) and in Iraq (until 2003), and it is currently being used in Mexico (USGS 2016, TSI 2016). According to the Polish law (Act 1995) and the adopted development plans, former extraction areas are subject to reclamation and mostly to reforestation (Pietrzykowski 2014). In reclaimed post-mining areas, forest tree species are introduced into conditions which are different from natural habitats, and technosols are frequently unable to harmoniously meet the nutritional requirements of trees (Mudrák et al. 2010; Pietrzykowski et al. 2013). Despite reclamation treatments, vegetation in such areas often exists in conditions of environmental stress caused by acidification and contamination of soil and groundwater with sulfur, and also disturbed quantitative relations of biogenic elements (Likus-Cieślik et al. 2017). For this reason, research in these conditions supplements information about the response of trees to physiological stress and the operation of the emerging forest ecosystems in changing environmental conditions (Pietrzykowski et al. 2013, 2014).

Research on mineral nutrition of plants in the conditions of reclaimed post-industrial areas is one of the most important factors in assessing the growth of the restored ecosystems. The aim of this study was to assess the supply of mineral to trees used in reforestation (birch and pine) and to wood small-reed which is dominant in the undergrowth and which appears by way of succession in microhabitat conditions forming in former sulfur extraction areas of the Jeziórko mine where there is excessive concentration of sulfur in the soil. The results are also relevant in an assessment of mutual relations in soils and plant tissues and to determine the tolerance ranges of sulfur concentrations.

## Material and methods

### Study site

**Fig. 1:**
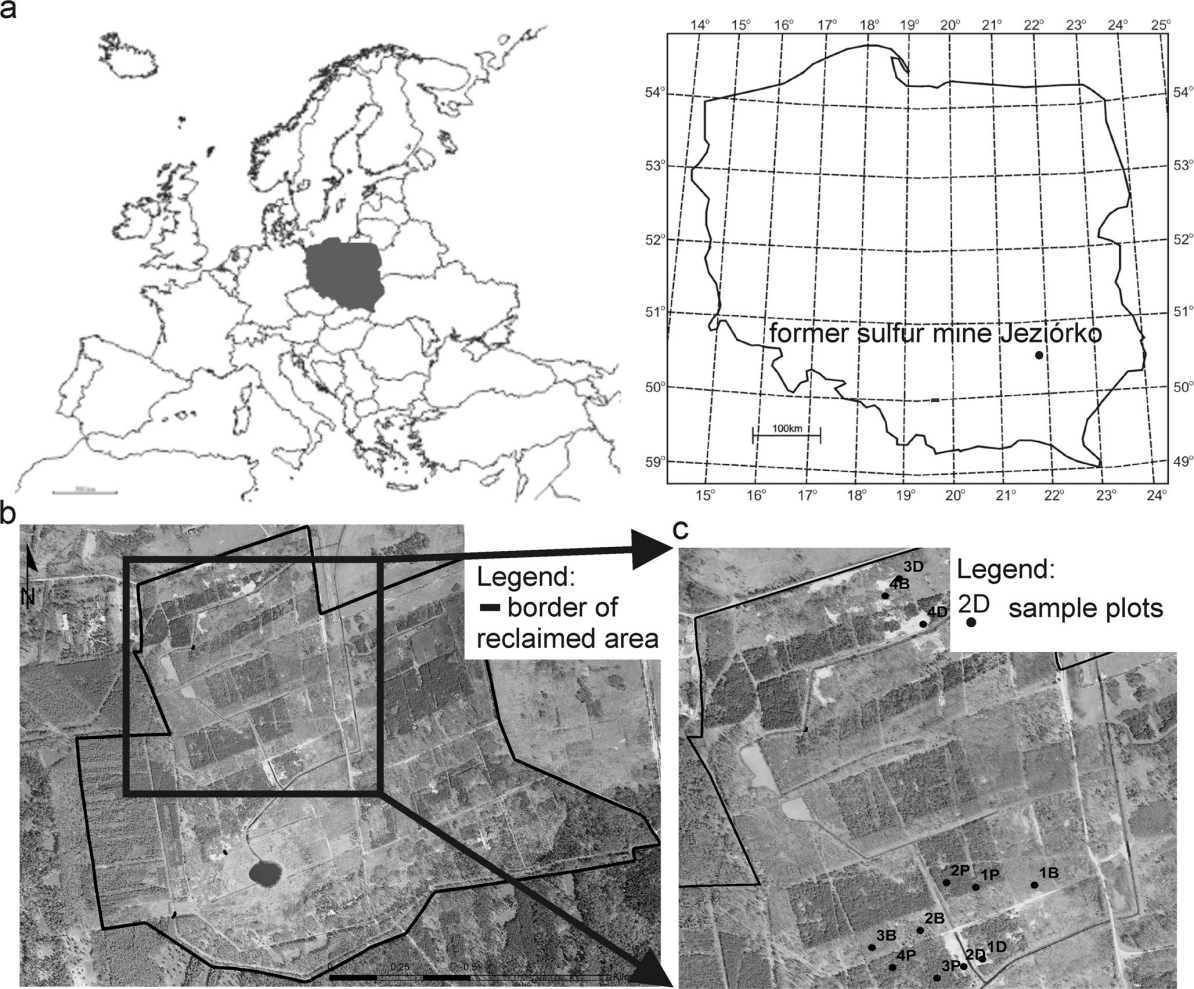
Location of study site (**a**), investigated part of the Forest District with reclaimed ecosystem (**b**), and sample plot location (**c**)

The study was conducted on reclaimed and reforested mining areas of the former Jeziórko sulfur mine (FSMJ) (Southern Poland, 50° 32′ 34 N, 21° 47′ 46 E) (Fig. 1a). The region has an average annual temperature of +8.2 °C (−1.6 °C in January and +18.7 °C in July) and average annual precipitation ranges from 550 to 650 mm. The growing season lasts from 200 to 220 days per annum.

Reclamation treatments of the area of the FSMJ started in 1993. Of over 2000 ha previously occupied by the mine, over 700 ha in total were reforested, of which 216.5 ha, where this research was conducted, are currently managed by Stale Forest District, Nowa Dęba Forest Inspectorate, State Forest National Forest Holding (Fig. 1b). Reclamation treatment included removing the mine infrastructure (such as mine wells, pipelines, and access roads), improvements to hydrographic conditions and landscaping, pH neutralization of sulfurous and excessively acidic soils by liming (average 400–500 Mg ha^−1^), fertilization (70 kg ha^−1^ P_2_O_5_, 60 kg ha^−1^ K_2_O), and the sowing of grass seed (Likus-Cieślik et al. 2017). These treatments were followed by reforestation, mainly with 1-year-old Scots pine seedlings (*Pinus sylvestris* L.) and an admixture of silver birch (*Betula pendula* Roth), and boreal oak (*Quercusrubra* L.) (Likus-Cieślik et al. 2015).

### Sampling and laboratory analyses

In the first step, an inventory was made to determine the cover-abundance classes using screen vectorization and a 25-cm pixel orthophotomap (Likus-Cieślik et al. 2015). Three plot categories were determined, verified, and marked in the field: category D—degraded, i.e., potentially ineffectively reclaimed and unsuccessfully reforested plots with low cover-abundance of vegetation; category P—pine stands; and category B—birch stands successfully reforested. The study plots were located in the field using GPS measurements (Trimble Pathfinder ProXRS receiver) and geotagging photos (using a photointerpretation key). Within each of the determined categories, four plots (from 4 to 6 ares in size) were defined (4 replication for each category, i.e., 12 plots in total, with symbols to mark the progress of field work, Fig. 1c). A regular square grid (10 × 10 m) was laid out on each study plot; soil subsamples were collected at the intersection of each square from two horizons: 0–20 cm and 20–50 cm, i.e., a total from 18 to 24 samples per plot. Next, collective samples were made (for 0–20 and 20–50 cm horizons) to represent a given study plot. Soil characteristics were determined in the samples: pH in KCl 1 M, soil electrical conductivity (EC), total sulfur (St), soil organic carbon (SOC), and total nitrogen (Nt). At the same intersections of the grid (10 × 10 m) on each study plot, 1 m^2^ subplots were laid out, and vegetation inventory was conducted along with an assessment of cover-abundance using the Braun-Blanquet scale (Braun-Blanquet 1964). Reference data for a tree (layer a) inventory and measurements (d.b.h. and height ht) were provided in an earlier publication (Likus-Cieślik et al. 2015). In autumn 2016 foliage tissue, i.e., leaves (birch) and needles (pine, current P_c_, and 2-year-old needles P_c+1_) were collected from trees growing on reclaimed and reforested areas (categories P and B) as a composite sample from five sample trees of uniform SW expositions and the middle section of tree crowns on each sample plot.

At designated points on the study plots, composite samples were collected (one from each plot) of wood small-reed leaves (*Calamagrostis epigejos* (L.) Roth) that is an undergrowth species which occurred at each study plot.

Vegetation samples were taken to a laboratory and dried at a temperature of 65 °C. The samples were then ground and prepared to determine the content of the following macroelements: nitrogen (N) and sulfur (S) with LecoTruMac®—CNS, and K, P, Ca, and Mg following mineralization in a mixture of HNO_3_ and HClO_4_ acids (in a ratio of 3:1) using an ICP OES ICAP 6000 Series spectrophotometer (by Thermo Fisher Scientific). ICP-OES (inductively coupled plasma optical emission spectrometry) is standard analytical technique used for the detection of elements. It is a flame technique with a flame temperature in a range from 6000 to 10,000 K, and the intensity of this emission is indicative of the concentration of the element within the sample.

### Statistical analyses

The statistical calculations were performed using Statistica 12 software (StatSoft, Inc. 2014). Multivariate data analysis were applied (data clustering by hierarchical agglomerative using Ward method) for grouping data. The significance of differences was compared to the median soil properties such as pH, EC, the contents of St, Nt, and SOC between three categories (P, B, and D) and soil horizons (0–20 cm and 20–50 cm). The significance of differences in the contents of N, S, Ca, Mg, K, Na, and P in the foliage between categories (P and B) and differences between the content of these macronutrients between pine needles of different age (P_c_, P_c+1_) was also compared. Differences were tested by an ANOVA test, preceded by a Shapiro-Wilk test of normality, and Levene’s test of variance homogeneity. The ANOVA test was followed by multiple pairwise comparisons using Fisher’s LSD (least significant difference) post-hoc test. Based on the results of Fisher’s LSD test, homogeneous subsets were distinguished. In the case of a lack of normality distribution or variance homogenity, Kruskal-Wallis test was used. The Kruskal-Wallis test was followed by multiple comparisons of mean ranks for all groups post-hoc test. Based on the results of post-hoc test, homogeneous subsets were distinguished. The ANOVA test was used in case of normal distribution to assess significant difference between categories for Ca, Na in trees foliage, and S, N, P, K, Ca, Mg, Na in wood small-reed leaves. The Kruskal-Wallis test was used to assess significant difference between categories for soil parameters (pH, EC, St, Nt, SOC), and S, N, P, K and Mg in trees foliage. Potential correlations between sulfur concentration in tree foliage as well as in wood small-reed tissue and soil properties (pH, EC, St, SOC, Nt) were determined by Spearman rank correlation calculations (due to lack of normal distribution).

The correlation coefficient with the number of samples *n* = 12 for tree foliage, and *n* = 12 for wood small-reed tissues was calculated at probability *p* = 0.05. Correlations between the investigated soil properties and sulfur concentration in plant tissues were described with a regression equation.

## Results

### Basic soil parameters

Soil pH in KCl in category D was the lowest and ranged from extremely acidic 1.5 to slightly alkaline 7.1 (Table 1). In categories P and B, pH was more elevated than in category D. There were significant differences in pH between degraded categories (D) and birch stand (B), and degraded categories and pine tree stands (P). In categories P and B, significant differences in pH between 0–20 cm and 20–50 cm horizons were also found (Table 1).

**Table 1:** Basic soil characteristics

Elements			Category and soil horizon					
			D		P		B	
			0–20	20–50	0–20	20–50	0–20	20–50
pH	[μS cm^−1^]	Median (mean)	3.1^ac^ (3.8)	2.7^a^ (3.3)	7.0^b,*^ (6.0)	4.4^c,**^ (4.6)	7.0^b,*^ (6.7)	4.6^c,**^ (5.0)
		Range	1.7–7.7	1.5–7.1	2.5–7.8	2.4–7.1	1.7–9.0	2.7–8.0
EC		Median (mean)	1960^a^ (1885)	1730^a^ (1932)	162^b^ (396)	61^b^ (345)	164^b^ (707)	67^b^ (421)
		Range	55–5580	40–6500	10–1709	23–1600	37–5920	14–2230
St	[mg kg^−1^]	Median (mean)	9595^a^ (25303)	10949^a^ (24868)	639^b,*^ (2246)	50^b,**^ (943)	545^b^ (17610)	156^b^ (12718)
		Range	107–179,370	50–245,740	50–32,462	50–19,779	50–330,160	50–162,470
Nt	[%]	Median (mean)	0.02^a^ (0.02)	0.02^a^ (0.02)	0.05^b*^ (0.06)	0.03^a**^ (0.03)	0.04^b*^ (0.05)	0.01^a**^ (0.01)
		Range	bdl-0.08	bdl-0.07	0.01–0.11	bdl-0.12	bdl-0.12	bdl-0.06
SOC	[%]	Median (mean)	0.98^a^ (1.33)	0.89^a^ (1.14)	1.57^a*^ (1.36)	0.55^b**^ (0.75)	1.38^a*^ (1.80)	0.28^b**^ (0.60)
		Range	0.08–4.82	0.03–3.03	0.28–3.59	0.06–4.37	0.11–4.92	0.04–6.09

^a, b, c^Significant differences between categories, *, ** significant differences between soil horizon in category

*B* birch stand category, *P* pine stand category, *D* degraded category, *bdl* below detection level i.e., 0.01%

Soil EC was highly variable and ranged from 40 to 6500 μS cm^−1^ (the highest found in category D). EC was the lowest in the category P, but it was also highly variable and ranged from 10 to 1709 μS cm^−1^. The observed differences in EC between categories D and P, as well as D and B were significant, but there was no difference in EC between soil horizons (0–20 cm and 20–50 cm) (Table 1).

The highest St in soil was found in category D (Table 1). The lowest St content was found in category P soils (Table 1). As in the case of EC, the St content differentiated the investigated soils but did not differ between the 0–20 and 20–50 cm horizons within the individual categories suggesting a significant sulfur migration in the soil profiles (Table 1).

SOC content in category D was similar in 0–20 and 20–50 cm soil horizon. SOC in category P and B were higher in 0–20 cm soil horizon (Table 1). This is due to a greater accumulation of humus in the upper horizons of reclaimed forest soils. There were no differences in the content of SOC between the two categories (Table 1).

Nt contents in soils differed between the categories and between soil horizons in categories P and B (Table 1). The lowest Nt nitrogen content was reported in category D with low cover-abundance and poorly developed humus horizons.

### Tree and tree stand parameters—reference data of cover-abundance and community richness

According to tree stand reference data, the tree stands in this category displayed moderate density. The mean diameter at breast height (d.b.h.) for the pine (category P) was 10.6 cm, mean height (ht) was 10.0 m, the mean volume of trees (V) 0.93 m^3^ are^−1^, while the number of trees (N) averaged 1457 pcs ha^−1^. Tree stands in this category displayed maximum density. The mean d.b.h. for the birch (category B) was 7.8 cm, mean ht. was 8.3 m, mean V was 0.50 m^3^ are^−1^, mean N was 1558 pcs ha^−1^ (reference data Likus-Cieślik et al. 2015).

In category P, 15 species were reported in the undergrowth layer c including four species of tree seedlings as well as two species in the bryophyte layer d (Table 2). In category B, 11 species were found in the undergrowth layer c including two species of tree seedlings (Table 2). Individual occurrence of one species in the bryophyte layer d was reported (Table 2). In category D, seven species were found in the undergrowth layer c, including one species of tree seedlings, and two individual species in the bryophytes layer d (*Entodon schreberi* and *Pohlia nutans*) (Table 2).

**Table 2:** Vegetation occurring on the FJSM (divided into categories of pine stands P, birch stands B, and treeless degraded areas D)

Layer	Species	Category		
		B	P	D
Layer c	*Achillea millefolium*			+
	*Calamagrostis arundinacea*			+
	*Calamagrostis epigejos*	2	2	2
	*Carex sylvatica*	2	1	
	*Cladoniaceae*	+		
	*Corynephorus canescens*	+		
	*Hieracium pilosella*		+	+
	*Homogyne alpina*	+		
	*Juncus effusus*			+
	*Lotus corniculatus*		+	
	*Oenothera biennis*	+		
	*Plantago lanceolata*			+
	*Potentilla anserina*		+	
	*Pteridium aquilinum*	+	+	
	*Pyrola sp.*		+	
	*Senecio sylvaticus*	+	+	
	*Solidago canadensis*	+		
	*Taraxacum campylodes*		+	
	*Torilis japonica*		+	
	*Trifolium pratense*		+	
Seedlings	*Betula pendula*		+	
	*Quercus robur*	+	+	
	*Pinus sylvestris*	+	+	
	*Populus alba*		+	+
Layer d	*Entodon schreberi*	1	2	+
	*Dicranum scoparium*		+	
	*Pohlia nutans*			+

The Braun-Blanquet cover abundance scale was used

+ rare, with a very small degree of covers, *1* numerous individuals but not abundant, *2* range of cover from 5 to 25%, (Braun-Blanquet 1964)

Wood small-reed (*C. epigejos* (L.) Roth.) was the dominant species in all the areas in the layer of herbaceous plants, with the exception of one in category D. In category P, an average of 35% cover-abundance was reported in the undergrowth layer, in category B 74%, while in category D 26% with wood small-reed accounting for an average of 10% (P), 19% (B), and 22% (D).

### Tree foliage chemistry

Birch showed significantly higher concentrations of N, P, K, Ca, Mg, and Na in the leaves than Scots pine in the needles (Table 3), while there were no significant differences in the supply of nutrients in current year and 2-year-old pine needles (Table 3). Phosphorus (P) content in pine needles was from 1221.3 to 1454.3 mg kg^−1^ in Pc, from 970.6 to 1111.8 mg kg^−1^ in P_c+1_. P content in birch leaves ranged from 1695.8 to 2969.3 mg kg^−1^. Potassium (K) content in birch leaves ranged from 0.68 to 1.07%. K content was pine needles from 0.46 to 0.56% in P_c_, and from 0.49 to 0.53% in P_c+1_. Magnesium (Mg) in birch leaves ranged from 1040 to 2073 mg kg^−1^. Mg content in pine needles ranged from 645 to 766 mg kg^−1^ in P_c_, and from 688 to 752 mg kg^−1^ in P_c+1_. Sodium (Na) content in birch leaves ranged from 17.5 to 47.9 mg kg^−1^. Na content in pine needles was from below detection level (i.e., 0.1 mg kg^−1^) to 21.7 mg kg^−1^ in P_c_, and from 1.3 to 21.7 mg kg^−1^ in P_c+1_.

**Table 3:** The supply of macronutrients of birch and pine foliage (broken down into needles from 2 years) in sulfurous soils of FSMJ

Elements			Category and research material		
			B	P	
				P_c_	P_c+1_
N	[%]	Median (mean)	1.68^a^ (1.71)	1.07^b^ (1.12)	1.10^b^ (1.11)
		Range	1.58–1.89	1.05–1.30	1.05–1.81
P	[mg kg^−1^]	Median (mean)	2309.3^a^ (2320.4)	1358.3^b^ (1348.1)	1049.6^b^ (1045.4)
		Range	1695.8–2969.3	1221.3–1454.3	970.6–1111.8
Ca	[%]	Median (mean)	1.30^a^ (1.35)	0.32^b^ (0.33)	0.78^c^ (0.75)
		Range	1.15–1.63	0.25–0.44	0.52–0.93
Mg	[mg kg^−1^]	Median (mean)	1494.9^a^ (1525.7)	705.2^b^ (705.2)	710.6^b^ (715.0)
		Range	1039.9–2073.1	645.0–765.5	687.5–751.5
K	[%]	Median (mean)	0.72^a^ (0.80)	0.49^b^ (0.50)	0.49^b^ (0.50)
		Range	0.68–1.07	0.46–0.56	0.48–0.53
Na	[mg kg^−1^]	Median (mean)	37.1^a^ (34.9)	11.0^b^ (10.3)	8.5^b^ (10.0)
		Range	17.46–47.88	bdl.—19.1	1.3–21.74

^a, b^Significant difference between categories

*B* birch stand category, *P* pine stand category, *P*_*c*_ current year needles, *P*_*c+1*_ 2-year-old pine needles, *bdl* below detection level

In the case of Ca content, differences in pine and birch foliage and between P_c_ and P_c+1_ were significant (Table 3). As mentioned above, the highest calcium (Ca) content was found in birch leaves.

**Graph 1:**
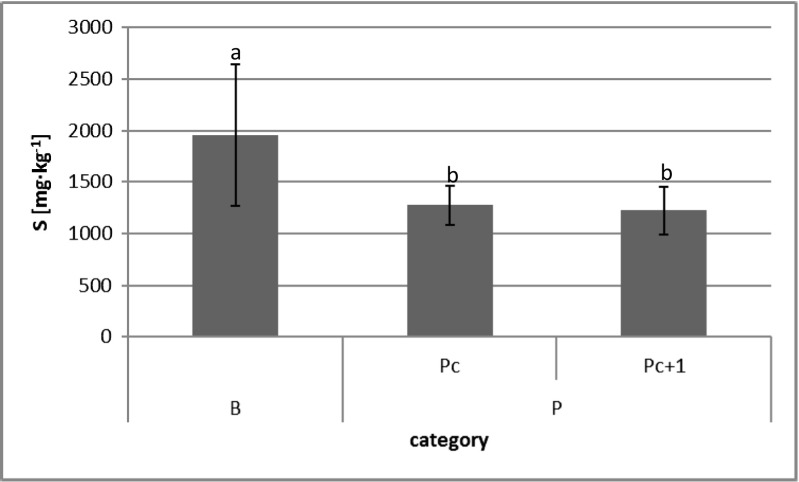
Sulfur concentration in tree (birch and pine) foliage with distinction into current year and 2-year-old needles in sulfurous soils of FSMJ

Like in the case of the mentioned basic macroelements, the highest sulfur (S) content was reported in birch leaves (mean 1954 mg kg^−1^). Differences between pine foliage years (P_c_ and P_c+1_) were not significant (Graph 1).

**Graph 2:**
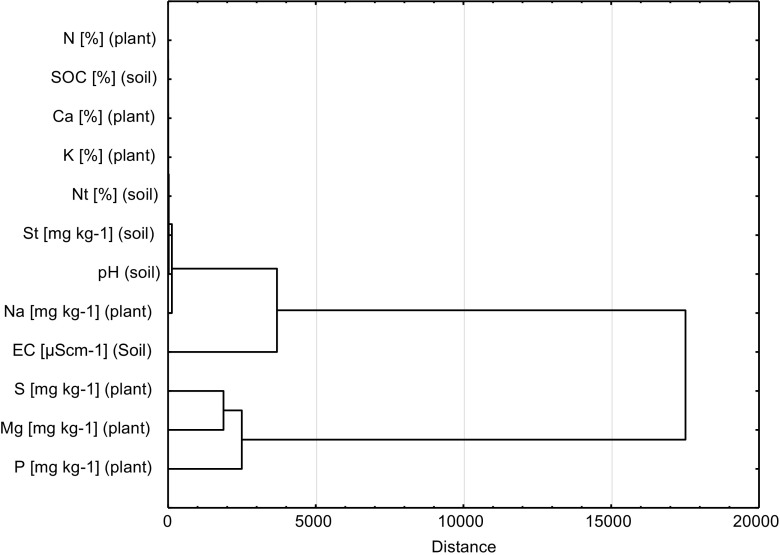
Plot of hierarchical tree (supply of macronutrients of birch, pine foliage, and soil characteristics)

Two main groups in tree foliage were found by data clustering process (Graph 2). The first group was composed of St concentration in soil, soil pH, soil EC, and Na concentration in plant (Graph 2).The second group was composed of S, Mg, and P concentration in plant (Graph 2). We also found that all these characteristics form one group but distance between data point is wide, so characteristics were weak correlated.

### *Calamagrostis epigejos* (wood small-reed) foliage chemistry

Wood small-reed occurring in the pine stand undergrowth displayed highest nutrient content, while the lowest was reported for the wood small-reed in treeless degraded areas (category D) (Table 4). The lowest N concentration in wood small-reed leaves which was significantly different from the pine stands (category P) occurred in category D and ranged from 0.40 to 0.72% (mean 0.62%) (Table 4).

**Table 4:** Nutrient content and N:P ratio in wood small-reed leaves occurring in designated categories in reforested areas of FSMJ

			Category		
			P	B	D
S	[mg kg^−1^]	Mean (median)	2249.1^a^ (2262.8)	1717.3^a^ (1689.1)	1778.5^a^ (2042.4)
		Range	2087.8–2383.0	1406.6–2084.4	790.6–2238.5
N	[%]	Mean (median)	0.93^a^ (0.97)	0.75^ab^ (0.73)	0.62^b^ (0.67)
		Range	0.71–1.09	0.56–0.99	0.40–0.72
P	[mg kg^−1^]	Mean (median)	1351.2^a^ (1381.1)	1132.5^a^ (1182.3)	904^a^ (739.3)
		Range	1235.3–1407.3	749.2–1416.3	519.3–1618.3
K	[%]	Mean (median)	0.95^a^ (0.87)	0.72^a^ (0.76)	0.57^a^ (0.57)
		Range	0.82–1.25	0.37–0.98	0.23–0.91
Ca	[%]	Mean (median)	0.34^a^ (0.33)	0.27^a^ (0.31)	0.22^a^ (0.24)
		Range	0.20–0.50	0.15–0.33	0.09–0.28
Mg	[mg kg^−1^]	Mean (median)	706.1^a^ (644.1)	485.5^ab^ (506.1)	363.9^b^ (390.6)
		Range	603.4–932.5	296.8–633.1	175.1–499.5
Na	[mg kg^−1^]	Mean (median)	25.0^a^ (15.8)	22.2^a^ (22.4)	11.3^a^ (5.9)
		Range	13.6–54.7	13.3–30.4	4.2–29.3
N:P ratio		Mean (median)	6.9	6.7	7.7

^a, b^Significant difference between categories

*B* birch stand category, *P* pine stand category, *D* degraded category

The content of phosphorus, potassium, calcium, and sodium in wood small-reed leaves growing in pine stands (category P), birch stands (category B), and degraded area (category D) was similar—there were no significant differences (Table 4).Only in the case of Mg content, the difference between the degraded category (D) and pine stands (P) was significant (Table 4).

Like in the case of the mentioned basic nutrients with the exception of Mg, differences in sulfur S content in wood small-reed leaves occurring in the designated categories were not significant. However, wood small-reed occurring in pine stand undergrowth (category P) contained most sulfur (mean 2249.1 mg kg^−1^), while the least amount was reported for wood small-reed occurring in category B (mean 1717.3 mg kg^−1^). Wood small-reed growing in degraded areas (in category D) mean contained 1778.5 mg kg^−1^ of S (Table 4).

**Graph 3:**
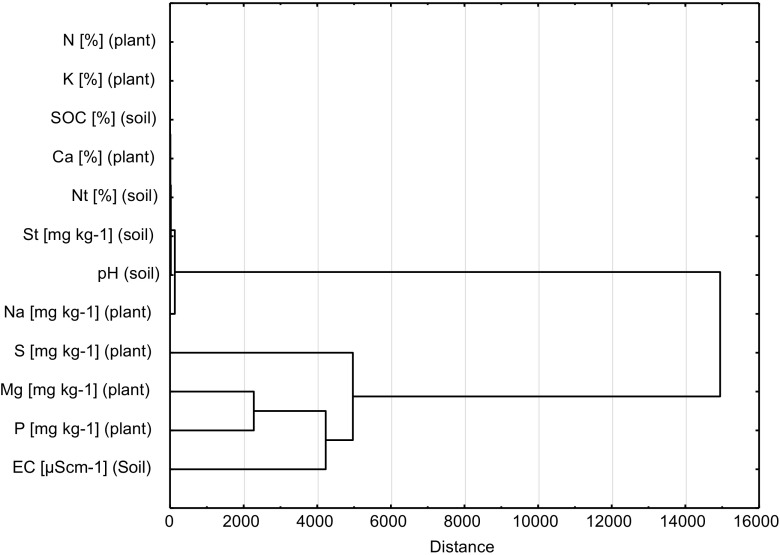
Plot of hierarchical tree (supply of macronutrients of wood small-reed leaves and soil characteristics)

We found two main groups in wood small-reed tissue in data clustering process (Graph 3). The first group was composed of soil properties as St, pH, and plant Na concentration. The second group was composed of S, Mg, P concentrations in plant and EC in soil (Graph 3).

### Relations between soil chemistry and sulfur content in plant tissue

**Graph 4:**
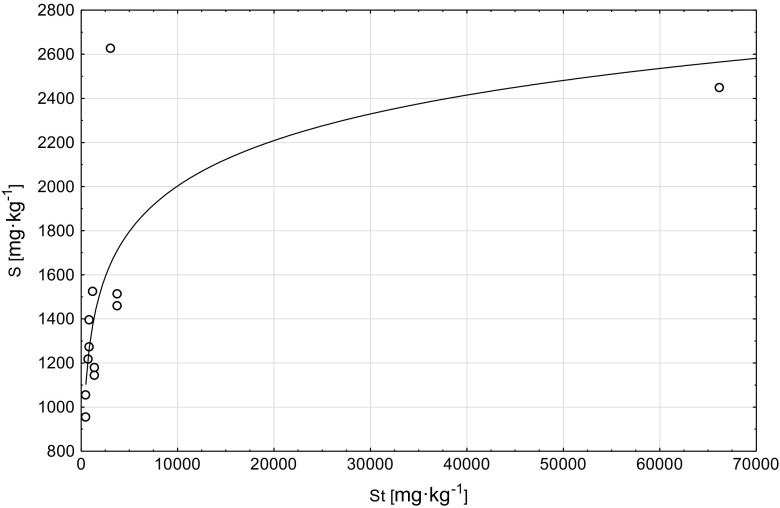
Scatter plots S (plant) vs St (soil)

**Graph 5:**
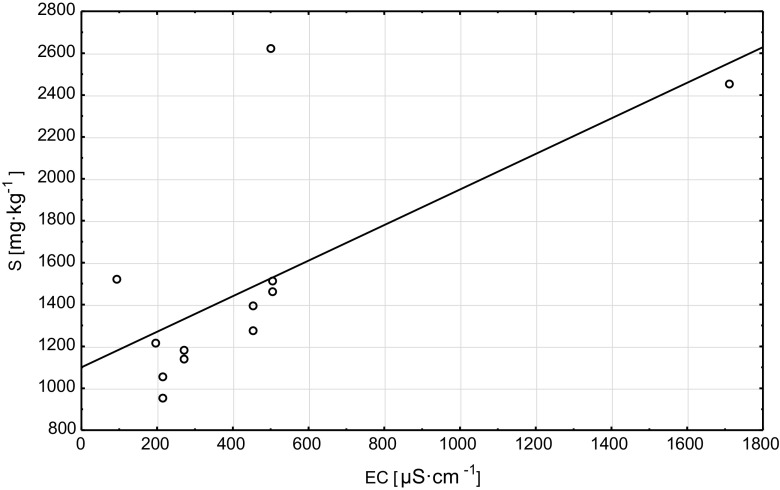
Scatter plots S (plant) vs EC (soil)

**Graph 6:**
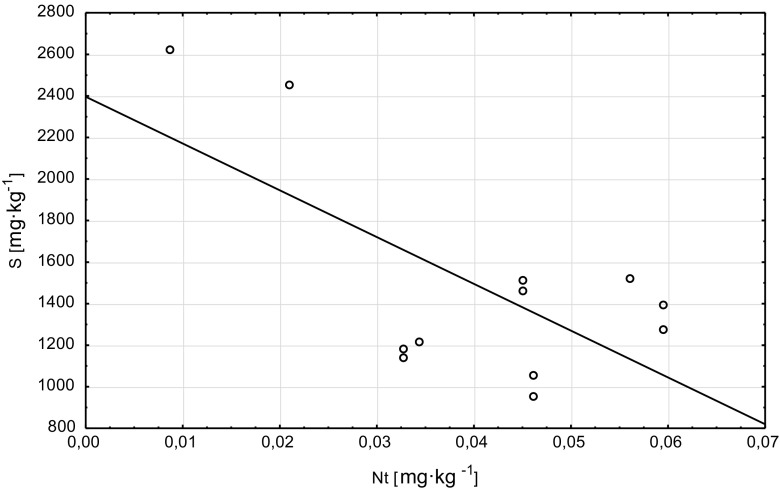
Scatter plots S (plant) vs Nt (soil)

A positive correlation was found between the sulfur (S) content in the tree foliage and the St content in soils (*r* = 0.61), these correlations are described by an equation of function *S* = 1362.33 + 0.02·St (Graph 4). Sulfur content in the tree foliage also significantly correlated with EC (*r* = 0.68; *S* = 1099.88 + 0.85·EC function equation, Graph 5) and Nt content in the soils (*r* = −0.66) (*S* = 2396.1–22,528.96·N function equation, Graph 6).

No correlation was found between sulfur content in wood small-reed tissues and the remaining soil parameters.

## Discussion

Based on data from an analysis of dry needle weight, it may be concluded that tree supply in nutrients Ca, Mg, and K is suitable in the investigated soils, while nitrogen (N) is a deficit nutrient for the trees occurring in the investigated areas. According to Baule and Fricker (1973), for forest trees to grow well, phosphorus content must amount to 0.12–0.13% and nitrogen to 1.2–1.3%. Lower content than the data provided by Baule and Fricker (1973) was reported for phosphorus in pine needles P_c+1_ and N content in current year and 2-year-old needles. The content of biogenic elements in the foliage of the investigated tree species is within the range provided by ICP-Forest (Stefan et al. 1997) as excellent element content. With regard to the nutrition of pine and birch trees on reclaimed land according to Heinsdorf (1999) from Lusatian Mine District (Eastern Germany), tree supply in N in the investigated former sulfur mine lands was exceedingly low for the pine (supply grade 1) and low for the birch (supply grade 2). According to the cited authors, the threshold P content for the birch was sufficient (supply grade 3), and good for the pine (supply grade 4), K content for the birch and pine was good (supply grade 4), and Mg content was sufficient (supply grade 3).

In assessing forest tree stand nutrition, it is also important to evaluate the proportions between macronutrients (Baule and Fricker 1973). The N:P ratio in pine needles was 8.3 for P_c_, 10.6 for P_c+1_, and 7.6 in birch leaves. Research on the content of macroelements in the pine needles on the post-mining areas in Poland was also conducted by Pietrzykowski et al. (2013). The N:P ratio in pine needles obtained by the authors in the former Piaseczno sulfur mine was 10, and so research in FSMJ yielded similar values as in the cited work. Pietrzykowski et al. (2013) found no significant differences between the N:P in mine land and in the control (managed stands in natural habitats).

*C. epigejos* is highly tolerant to abiotic stress factors (Holub et al. 2012). *C. epigejos* is a dominant species in post-mining lands in initial successional stage in Central Europe (Mudrák et al. 2010; Roubíčková et al. 2012). It outcompetes (mainly due to clonal propagation) most other plant species occurring in soils rich in nitrogen (Mudrák et al. 2010; Roubíčková et al. 2012). In post-mining areas, *C. epigejos* occurs in monospecific stands mostly from 5 to 45 years old (Roubíčková et al. 2012). Our research indicates that *C. epigejos* is tolerant to high S concentration in the soil. In areas with increased sulfur concentration (D), higher amounts of this element were not reported in aboveground tissue parts of wood small-reed compared to areas in pine and birch stands which displayed lower sulfur content in the soil. This phenomenon could be caused by neutralization with flotation lime sludge used in different doses during the initial reclamation phase on studied area. The flotation lime sludge dose depended on the sulfur content in the soil. If the sulfur content in a 20 cm soil layer did not exceed 3%, a dose of 400 to 500 Mg kg^−1^ of flotation lime was applied. In areas with higher sulfur content, additional mechanical removal of sulfur from the surface and an extra isolating layer of lime of about 10 cm was recommended (Gołda 2003). Spatial distribution of such places and concentration of sulfur in relationships to vegetation was presented in details by Likus-Cieślik et al. (2017). The authors have found that despite of high sulfur concentration (even 2500 mg kg^−1^) vegetation cover-abundance was in some cases even 70%. In such places, simultaneously soil pH was above 4.0, because of neutralization effect. In Alberta (Canada), the major producer of sulfur a by-product of sour natural gas processing in 80s of the twentieth century, complete loss of cover vegetation (mosses) and drastically decreases of shrubs at site less than 100 m from emitter at soil sulfur concentrations above 29,000 mg kg^−1^ and pH values below 3 were found (Cárcamo et al. 1998; Cárcamo and Parkinson 2001). Mentioned authors (Cárcamo and Parkinson 2001) have found that in greater distance from emitter vegetation was better developed, cover-abundance was higher (8.9 average plant richness·m^−2^ in area above 250 m from emitter); however, soil pH was higher (5.5–6.3). High cover vegetation in degraded area (22% out of 26% of the cover-abundance in the layer of herbaceous plants) and no increased uptake of sulfur by *C. epigejos* may be caused by high pH value. SO_2_ contained in acid deposition is also dangerous for plant existence. Acidic rain adversely affects agricultural plants and forests plants due to leaf damage from direct contact, the acidification of soils, and the reduction of nutrient availability (Guala et al. 2009; Liu et al. 2010). During an increase of acidity on springtime or summertime, plants are severely damaged, diminishing the efficiency of metabolic process and the survival capacity (Guala et al. 2009). By analyzing the results in FSMJ, it seems that the existing of vegetation and lack of reaction to excessive sulfur concentration in the areas on post-mining sulfur is conditioned by the liming in reclamation process. In areas with pine stands (category P), nitrogen content in aboveground parts of *C. epigejos* was similar to the content observed in natural habitats by Holub et al. (2012) in studies conducted in the Podyjí National Park (0.84–1.03% N and 1600–1700 mg kg^−1^ P). Wood small-reed growing in the undergrowth of birch stands (category B) and on degraded areas (D) showed lower nitrogen supply and increasing in all categories (birch, pine, and degraded) lower supply of phosphorus in relation to the cited data from research on natural habitats. The obtained nitrogen and phosphorus content is, however, lower that the optimum content for proper development of vegetation cited by Marcshner (2012) which amounts to 1–5% for N and P for 3000–5000 mg kg^−1^. This suggests that, as in most technosols on reclaimed areas, nitrogen and phosphorus may be deficit elements for vegetation, both trees and herbaceous plants of the undergrowth (Pietrzykowski et al. 2013).

## Conclusions

Studies have shown that trees introduced on former sulfur mine areas following properly carried out neutralization treatment (as reflected by higher soil pH compared to soils of treeless degraded categories) grow well and are sufficiently supplied with most nutrients with the exception of nitrogen and in some cases phosphorus. These elements, as in the majority of post-mining areas reclaimed to forest, are a minimum factor in tree stand mineral nutrition, but higher nutrient content including sulfur were reported for the birch.

While in the case of the investigated tree species occurring in sulfurous soils which however are not excessively phytotoxic, linear correlations between the soil and plants were found, in the case of wood small-reed, *C. epigejos* occurring in all categories, including highly sulfurous soils with pH of about 4.0, these correlations were not linear. Wood small-reed showed no increased uptake of sulfur and perhaps uses the strategy of blocking pollutant uptake from the soil (true exclusion or blocking of elements). Undoubtedly, it is a species resistant to environmental stress conditions and may be a plant used in the reclamation and remediation treatments of such soils typically displaying elevated salinity (EC) and sulfur content.

## Abbreviations

FSMJ: former sulfur mine Jeziórko
P: pine stand category, successfully reforested
B: birch stand category, successfully reforested
D: degraded category, i.e., ineffectively reclaimed and unsuccessfully reforested plots, with low cover-abundance or complete lack of vegetation
P_c_: current year needles
P_c+1_: 2-year-old needles
EC: soil electrical conductivity
St: total sulfur
Nt: total nitrogen
SOC: soil organic carbon
d.b.h.: tree diameter at a breast height of 1.3 m
ht.: mean height
V: total volume of tree
N: number of trees on 1 ha area
